# Effects of high-intensity swimming training on the bones of ovariectomized rats

**DOI:** 10.20463/jenb.2016.09.20.3.6

**Published:** 2016-09-30

**Authors:** Taewoong Oh, Sakura Tanaka, Tatsuki Naka, Shoji Igawa

**Affiliations:** 1 Dean of the College of Sports Sciences, Yongin University, Yongin-Si Republic of Korea; 2 Graduate School of Health Science, Matsumoto University, Nagano Japan; 3 Faculty of Wellness, Shigakkan University, Aichi Japan

**Keywords:** High-intensity exercise, Swimming training, Bone, Ovariectomized rat

## Abstract

**[Purpose]:**

This study was performed to assess the effects of high-intensity intermittent swimming training(HIT) on bone in ovariectomized rats.

**[Methods]:**

Six-week-old female Sprague-Dawley rats were randomly assigned to either sham operation or bilateral ovariectomy. After surgery, they were divided into the following four groups: 1) sham-operated sedentary (S), 2) sham-operated exercise training (SE), 3) OVX sedentary (O), 4) OVX exercise training (OE) 5) OVX given 17β-estradiol (OE2) and 6) OVX exercise training and given 17β-estradiol (OEE). SE, OE and OEE rats were used extremely high-intensity swim exercise. The rats repeated fourteen 20-s swimming bouts with a weight equivalent to 14, 15, and 16% of body weight for the first 5, the next 9, and the last 5 days, respectively. Between exercise bouts, a 10-s pause was allowed. HIT was originally designed as an exercise method; a method that very quickly induces an increase in the maximum oxygen intake (Tabata I et al., 1996). OEE and OE2 rats were subcutaneously injected ethanol with 25μg/kg body weight 17β-estradiol 3 times per week.

**[Results]:**

Bone strength, bone mineral density and trabecular bone parameters were measured after a 8-weeks experimental period. Bone strength was significantly higher in the SE, OE, OE2 and OEE group compared with the O group. BV/TV was significant increase in the SE, OE groups compared with the O group. BMD showed no difference in the OE group compared with the O group.

**[Conclusion]:**

This study demonstrate some beneficial effects of postmenopausal osteoporosis of high-intensity intermittent swimming training on bone structure and strength.

## INTRODUCTION

Postmenopausal osteoporosis is a serious problem in elderly women and is characterized by a decrease in bone mass, leading to fractures and imbalanced bone turnover^1, 2^. Achieving a high peak bone mass during childhood and adolescence and the suppression of bone loss due to aging are important factors influencing osteoporosis.

Ovariectomized rats present rapid loss of cancellous bone mass and strength. These bone loss features mimic the bone changes following oophorectomy or menopause in humans^3^. Although the presence of osteoporosis is determined primarily by genetic factors^4^, research has shown that exercise and nutrition may help in the prevention of the osteoporosis. However, the mechanism by which exercise exerts beneficial effects on the bones is not well understood.

Several studies have reported the effects of swimming training on the bones. In general, subjecting the skeleton to significantly less than the habitual skeletal loads results in a decrease in bone mass. Therefore, previous studies reported that it is difficult to prevent bone loss by swimming training alone^5, 6^. However, other studies demonstrated the beneficial effects of swimming exercise on bone structure, turnover, and strength in ovariectomized rats^7, 8^. These studies showed the effects of swimming training on the bones in relation to the mechanical stress on bones caused by muscular contraction.

In many of the previous studies that analyzed the effect of running exercise and swimming training on bones, the length of time in which the exercise was performed was relatively long. Exercises for the prevention of other diseases, aside from bone diseases, also requires a considerable length of time. However, in recent years the theory that short bursts of exercise can improve physical endurance at the same level or greater than longer exercise sessions^9, 10^ has been reappraised. The Tabata Protocol is high-intensity training (HIT) consisting of 8 20-second sets of ergometer exercises, which leaves the body exhausted by the 8th set. HIT hones the body’s two fuel systems (aerobic and anaerobic) simultaneously to the greatest extent possible, over a short period of time. Terada *et al*. found an increase in glucose transporter type 4 (GLUT-4) content in the skeletal muscle of male Sprague-Dawley rats after a short time of HIT^11^. This increase caused an intracellular energy deficiency because of the muscle contraction performed during the HIT, which led to fatigue and was thought to cause AMPKα activation and GLUT-4 translocation.

In the present study, we hypothesized that brief periods of HIT in combination with an estrogen supplement would have a beneficial effect on the bones. The purpose of the present study was to examine the effects of short periods of high-intensity swimming training (HIST) alone and in combination with estrogen replacement therapy on the bones of ovariectomized rats.

## METHODS

### Animals

We used 6-week-old female Sprague-Dawley rats (Crea Japan, Tokyo, Japan) with an initial body weight ranging from 130 to 160 g for this study. All animals were housed in a room with the lights on from 8:00 AM to 8:00 PM and were maintained on a libitum diet of standard chow and water. The room temperature was maintained at 22°C. All animal experiments were conducted in accordance with the ethical standards of the institutional Animal Care and Use Committee of the University of Matsumoto. The rats were randomly assigned to either the sham operation (n = 12) or bilateral ovariectomy (n = 45). 6 days after surgery, they were divided into the following 6 groups: 1) sham-operated and sedentary (S, n = 6); 2) sham-operated and exercise trained (SE, n = 6); 3) ovariectomized and sedentary (O, n = 10); 4) ovariectomized and exercise trained (OE, n = 11); 5) ovariectomized and given 17β-estradiol (OE2, n = 6); and 6) ovariectomized, exercise trained, and given 17β-estradiol (OEE, n = 6).

### Training protocol and medication

OE and OEE rats underwent extreme HIST. The swimming exercise was performed in a barrel (540×530×660 mm) filled with water at 35°C (50-cm deep). During the initial 2 days, all rats were adjusted to the swimming exercise without any load. During the next 10 days, all rats were exercised with a weight equivalent to 2%~10% of their body weight. After that, the rats repeated fourteen 20-second swimming bouts with a weight equivalent to 14, 15, and 16% of their body weight for the first 5, the next 9, and the last 5 days, respectively. Between exercise bouts, a 10-second pause was allowed. OEE rats and OE2 rats were subcutaneously injected with ethanol with 25 μg/kg 17β-estradiol (Sigma-Aldrich Japan G.K., Tokyo, Japan) 3 times per week.

### Tissue collection

At the end of the experiment, the rats were fasted for 12 h and then were anesthetized with an intraperitoneal injection of pentobarbital sodium (5 mg/100 g of body weight) and sacrificed. Blood samples were collected to assay the biochemical parameters. The tibia, femur, visceral fat, and uterus were removed, and the wet mass was measured. After careful removal of all the soft tissue, the femur and tibia were fixed with 70% ethanol and stored at 4°C until the measurements were performed.

### Measurement of bone mineral density (BMD) and bone strength

BMD in the right femur and tibia were measured with dual-energy X-ray absorptiometry (DPX-L, Lunar, Madison, WI, USA). Subsequently, in the same bones, fracture strength and fracture energy (AGS-100 D, Shimadzu Corp., Kyoto, Japan) were measured using the breaking test of the 3-point support of the bone strength.

### Micro-computed tomography (Micro-CT)

Images of the left tibia were taken using the scanXmate- A080 (Comscan Co. Ltd., Kanagawa Prefecture, Japan), and a 3-dimensional analytic device was used to find the index values of the bone structure of the tibia cancellous bone. Regarding the images of the tibia, the longitudinal axis of the tibia was aligned with the axis of revolution, and the focus was set on a point eight grid-cells away from the epiphyseal line. A total of 480 continuous cross-sectional images were taken under the following conditions: tube voltage, 37.5 kV; tube current, 250 μA; matrix diameter, 12×480; projection, 600 frames; and the number of scans was 4. The parameters for the analysis were as follows: bone volume/tissue volume (BV/TV), trabecular thickness (Tb.Th), trabecular number (Tb.N), trabecular separation (Tb.Sp), and trabecular bone pattern factor (TBPf).

### Serum insulin concentration

To measure the concentration of serum insulin, we used a rat insulin measuring kit (Morinaga Institute of Biological Science, Inc., Yokohama, Japan). 95μL of guinea- pig anti-insulin serum and 5 μL of serum were added into each well of an antibody-coated plate. The plate was heated at 4°C for 16–20 hours and left to stand. Each well was then cleansed 3 times using a cleaning solution, 100 μL of enzyme-labeled anti-guinea-pig immunoglobulin G (IgG) antibody solution was added, and the plate was left to stand at room temperature for 1 hour. After cleansing the wells 5 more times with the cleaning solution, a reaction was instigated by adding 100 μL of E enzyme substrate solution to each of the wells and leaving the plate at light-shielded room temperature for 30 minutes. The reaction was then stopped by adding 100 μL of F reaction- stopping solution into each well, and the absorbance was measured using a spectrophotometer set at 450 nm (Infinite F200, Tecan Japan Co., Ltd., Kawasaki, Japan).

A standard curve insulin solution was used to calculate the calibration curve, which was then used to determine the serum insulin concentration (ng/mL).

### Serum Gla-osteocalcin concentration

To measure the serum Gla-osteocalcin concentration, we used a rat osteocalcin EIA kit (Biomedical Technologies, Stoughton, MA, USA). 25 μL of serum diluted 10- fold with a sample buffer were added to each well of a 96-well plate. 100 μL of osteocalcin antiserum were also added to each well, and then the plate was heated at 37°C for 2.5 hours. Each well was cleansed 5 times with a wash buffer. 100μL of BT-495 (donkey anti-goat IgG peroxidase) were then added to each well, and the plate was left to stand at room temperature for 1 hour. After cleansing the wells 5 more times with the wash buffer, a 100μL mixture of peroxidase substrate TMB and hydrogen peroxide solution was added into each well, and then, after 30 minutes, the plate was left standing at light-shielded room temperature. After adding 100 μL of stop solution to each well to stop the coloring reaction, absorbance was measured using a spectrophotometer set at 450 nm. An osteocalcin reference solution was used to calculate the calibration curve, which was then used to determine the serum Gla-osteocalcin concentration (ng/mL).

### Serum Glu-osteocalcin concentration

To measure the Glu-osteocalcin concentration in blood, a rat Glu-osteocalcin high sensitive EIA Kit (Takara Bio Inc., Shiga, Japan) was used. 100 μL of serum were added into each well of an antibody plate, and then left at room temperature for 1 hour to instigate a reaction. After the reaction, each well was cleansed 3 times using phosphate buffered saline, which contained 0.1% Tween-20, 100 μL of labeled antibody solution were added into each well, and the plate was left to stand at room temperature for 1 hour. The wells were then cleansed with the phosphate buffered saline 4 times. The reaction was instigated for 15 minutes by adding 100 μL of substrate solution, and then the reaction was stopped by adding 100 μL of reaction- stopping solution and mixing it well. Absorbance was then measured using a spectrophotometer set at 450 nm.

### Statistical analysis

All data are shown as mean values ± standard deviation. One-way analysis of variance was conducted, and Tukey’s honest significant difference method was used to determine statistically significant differences between groups.

## RESULTS

### Body mass and composition (Table 1).

Body mass of the rats 8 hours after the trial: The O and OE rats showed significantly higher values than the S rats (*p*<0.05). The OE, OE2, and OEE rats showed significantly lower values than the O rats (*p*<0.001). The OEE and OE2 rats showed significantly lower values than the OE rats (*p*<0.001).

Food intake per day: There were no significant differences between the groups.

Uterine weight per kilogram of body weight 8 weeks after the trial: The O and OE rats showed significantly lower values than the S and SE rats (*p*<0.001). The OE2 and OEE rats showed significantly higher values than the O and OE rats (*p*<0.001).

Fat weight per kilogram of body weight 8 weeks after the trial: The SE, OE, OE2, and OEE rats showed significantly lower values than the O rats (*p*<0.05). In addition, the OE2 and OEE rats showed significantly lower values than the OE rats (*p*<0.05).

**Table 1.** Body mass and composition

	S n=6		SE n=6		O n=10		OE n=11		OE2 n=6		OEE n=6	
	Mean	SE	Mean	SE	Mean	SE	Mean	SE	Mean	SE	Mean	SE
Final wt (g)	296.7	10.96	289.3^c^^d^^f^	6.05	366^a^^b^^d^^e^^f^	8.87	333.9^a^^b^^c^^e^^f^	6.68	273.3^c^^d^	3.62	242.3^a^^b^^c^^d^	9.94
Food intake (g/day)	17.4	0.62	15.8	0.54	19.0	0.97	17.3	0.50	16.2	0.89	16.7	1.80
UW (g)	0.62^c^^d^	0.06	0.64^c^^d^	0.10	0.13^a^^b^^e^^f^	0.01	0.10^a^^b^^e^^f^	0.01	0.62^c^^d^	0.04	0.60^c^^d^	0.04
UW/kg wt	0.21^c^^d^	0.02	0.23^c^^d^	0.03	0.04^a^^b^^e^^f^	0.00	0.03^a^^b^^e^^f^	0.00	0.24^c^^d^	0.01	0.25^c^^d^	0.03
FW (g)	8.92^c^	1.94	6.79^c^	0.81	14.54^a^^b^^d^^e^^f^	1.37	8.77^c^^f^	1.01	5.39^c^^d^	0.93	2.92^c^^d^	1.05
FW/kg wt	3.01^f^	0.61	2.44^c^	0.26	4.10^b^^d^^e^^f^	0.33	2.73^c^^f^	0.29	2.07^c^	0.35	1.24^a^^c^^d^	0.38
Soleus/kg wt	0.04	0.00	0.04	0.00	0.03	0.00	0.04	0.01	0.04	0.00	0.04	0.00

^a^p < .05 vs S

^b^p < .05 vs SE

^c^p < .05 vs O

^d^p < .05 vs OE

^e^p < .05 vs OE2

^f^p < .05 vs OEE

S, sham-operated and sedentary; SE, sham-operated and exercise trained; O, ovariectomized and sedentary; OE, ovariectomized and exercise trained; OE2, ovariectomized and given 17β-estradiol; OEE, ovariectomized, exercise trained, and given 17β-estradiol; Wt, weight; UW, uterine weight; FW, fat weight

### Bone volume, BMD, and breaking strength of the femur and tibia (Table 2 and Fig. 1)

Femur weight per kilogram of body weight: The O rats showed significantly lower values than the other rat groups (*p*<0.005). The OEE rats showed significantly higher values than the S, O, OE, and OE2 rats (*p*<0.05).

Proximal tibia weight per kilogram of body weight: The O rats showed significantly lower values than all the other rat groups (*p*<0.001). The OEE rats showed significantly higher values than the S, O, and OE rats (*p*<0.001).

Femur BMD per kilogram of body weight: The S, SE, OE2, and OEE rats showed significantly higher values than the O and OE rats (*p*<0.001). In addition, the OE2 rats showed significantly higher values than the O and OE rats (*p*<0.05).

Tibia BMD per kilogram of body weight: OEE rats showed significantly higher values than the S, SE, O, and OE rats (*p*<0.05). In addition, the OE2 rats showed significantly higher values than the O and OE rats (*p*<0.05).

Femur breaking strength: SE, OE, OE2, and OEE rats showed significantly higher values than the O rats (*p*<0.05).

Tibia breaking strength: SE, OE, OE2, and OEE rats showed significantly higher values than the O rats (*p*<0.05). SE and OE rats showed significantly higher values than the S rats (*p*<0.01).

**Figure 1.**
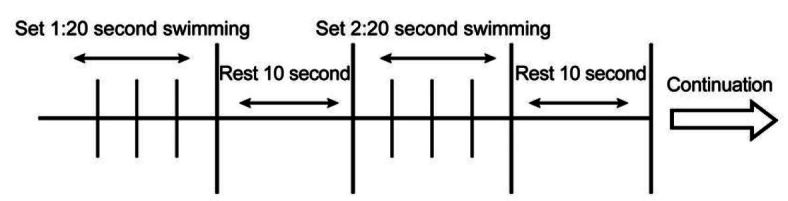
High-intensity swimming training protocol Number of repetitions per set: 14. The rats performed 20-s swimming bouts with a weight of a weight equivalent to 14, 15, and 16% of body weight for the first 5, the next 9, and the last 5 days, respectively.

**Table 2.** Bone weight, bone mineral density (BMD), and breaking strength of the femur (F) and tibia (T)

	S n=6		SE n=6		O n=10		OE n=11		OE2 n=6		OEE n=6	
	Mean	SE	Mean	SE	Mean	SE	Mean	SE	Mean	SE	Mean	SE
Femur/kg wt (g)	0.27^c^^f^	0.01	0.30^c^	0.01	0.23^a^^b^^d^^e^^f^	0.00	0.27^c^^f^	0.01	0.30^c^^f^	0.01	0.33^a^^c^^d^^e^	0.01
Tibia/kg wt (g)	0.22^c^^f^	0.01	0.25^c^	0.01	0.19^a^^b^^d^^e^^f^	0.00	0.23^c^^f^	0.00	0.25^c^	0.01	0.27^a^^c^^d^	0.01
F-BMD/kg wt (mg/cm2/kg)	0.08^c^	0.00	0.09^c^^d^	0.00	0.07^a^^b^^e^^f^	0.00	0.07^b^^e^^f^	0.00	0.09^c^^d^	0.00	0.10^c^^d^	0.01
T-BMD/kg wt (mg/cm2/kg)	0.06^f^	0.01	0.07^f^	0.01	0.05^e^^f^	0.00	0.05^e^^f^	0.00	0.08^c^^d^	0.00	0.10^a^^b^^c^^d^	0.01
F-breaking strength (kg*f)	6.89	0.34	7.39^c^	0.26	5.69^b^^d^^e^^f^	0.15	7.22^c^	0.26	7.76^c^	0.40	7.58^c^	0.58
T-breaking strength (kg*f)	4.83^b^^d^	0.30	6.10^a^^c^	0.20	4.11^b^^d^^e^^f^	0.14	6.14^a^^c^	0.25	5.24^c^	0.22	5.79^c^	0.26

^a^p < .05 vs S

^b^p < .05 vs SE

^c^p < .05 vs O

^d^p < .05 vs OE

^e^p < .05 vs OE2

^f^p < .05 vs OEE

S, sham-operated and sedentary; SE, sham-operated and exercise trained; O, ovariectomized and sedentary; OE, ovariectomized and exercise trained; OE2, ovariectomized and given 17β-estradiol; OEE, ovariectomized, exercise trained, and given 17β-estradiol; Wt, weight

### Bone metabolism and serum insulin concentration (Table 3)

Serum Gla-osteocalcin concentration: The OEE rats showed significantly lower values than the S, SE, O, and OE rats (*p*<0.01). In addition, the OE2 rats showed significantly lower values than the S, O, and OE rats (*p*<0.05). The SE rats showed significantly lower values than the O rats (*p*<0.05).

**Figure 2.**
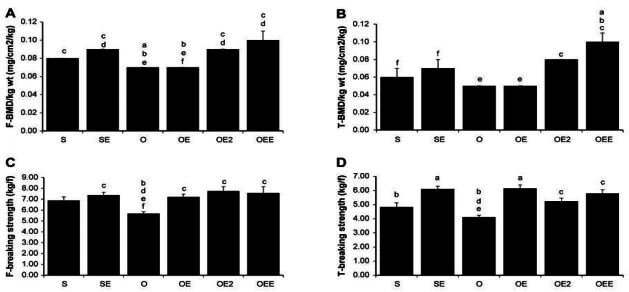
Bone mineral density (BMD) and bBreaking strength of femur and tibia Values are means ± SE for per groups. (A) = femur BMD; (B) = tTibia BMD; (C) = femur breaking strength; (D) = tTibia breaking strength. a p <.05 vs S, b p <.05 vs SE, c p <.05 vs O, d p <.05 vs OE, e p <.05 vs OE2, f p <.05 vs OEE. S, sham-operated and sedentary; SE, sham-operated and exercise trained; O, ovariectomized and sedentary; OE, ovariectomized and exercise trained; OE2, ovariectomized and given 17β-estradiol; OEE, ovariectomized, exercise trained, and given 17β-estradiol.

**Figure 3.**
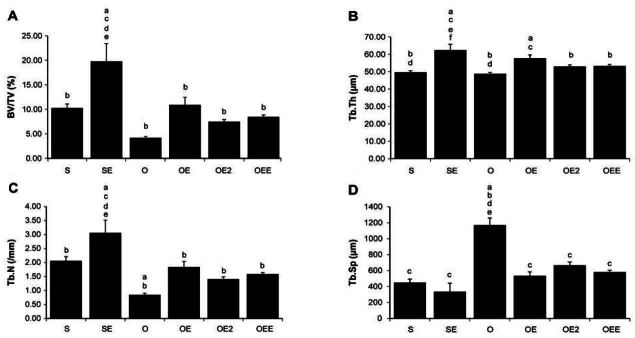
Micro-computed tomographyCT of the proximal tibia Values are means ± SE for per groups. (A)= BV/TV; (B)B= TB.Th; (C)= Tb.N; (D)= Tb.Sp. a p <.05 vs S, b p <.05 vs SE, c p <.05 vs O, d p <.05 vs OE, e p <.05 vs OE2, f p <.05 vs OEE. S, sham-operated and sedentary; SE, sham-operated and exercise trained; O, ovariectomized and sedentary; OE, ovariectomized and exercise trained; OE2, ovariectomized and given 17β-estradiol; OEE, ovariectomized, exercise trained, and given 17β-estradiol; BV/TV bone volume/tissue volume; Tb.Th, trabecular thickness; Tb.N, trabecular number; Tb.Sp, trabecular separation.

Serum Glu-osteocalcin concentration: The S, SE, OE2, and OEE rats showed significantly lower values than the O rats (*p*<0.01). The S, SE, OE2, and OEE rats showed significantly lower values than the OE rats (*p*<0.001).

Serum Gla/Glu-osteocalcin concentration: there were no significant differences between the groups.

Serum insulin concentration: there were no significant differences between the groups.

**Table 3.** Biochemical markers of bone metabolism and insulin levels

	S n=6		SE n=6		O n=10		OE n=11		OE2 n=6		OEE n=6	
	Mean	SE	Mean	SE	Mean	SE	Mean	SE	Mean	SE	Mean	SE
Gla-osteocalcin	39.08^e^^f^	6.80	37.18^c^^f^	1.69	52.11^b^^e^^f^	1.78	47.39^e^^f^	3.43	21.27^a^^c^^d^	2.73	15.79^a^^b^^c^^d^	0.86
Glu-osteocalcin	57.71^c^^d^	7.69	61.99^c^^d^	9.77	116.57^a^^b^^e^^f^	6.00	121.52^a^^b^^e^^f^	12.30	28.94^c^^d^	3.73	26.29^c^^d^	2.88
Gla/Glu-osteocalcin	0.79	0.22	0.68	0.12	0.45	0.02	0.41	0.04	0.66	0.06	0.64	0.08
Insulin (μg/L)	1.14	0.32	0.55	0.18	3.26	1.46	1.41	0.21	2.27	0.50	1.87	0.61

^a^p < .05 vs S

^b^p < .05 vs SE

^c^p < .05 vs O

^d^p < .05 vs OE

^e^p < .05 vs OE2

^f^p < .05 vs OEE

S, sham-operated and sedentary; SE, sham-operated and exercise trained; O, ovariectomized and sedentary; OE, ovariectomized and exercise trained; OE2, ovariectomized and given 17β-estradiol; OEE, ovariectomized, exercise trained, and given 17β-estradiol

### Tibia cancellous bone parameters as revealed by Micro-CT(Table 4)

Regarding the structure of the tibia proximal cancellous bone, the BV/TV ratio of the SE rats was significantly higher than that of all the other rat groups (*p*<0.005). In addition, the OE rats showed significantly higher BV/ TV ratio than the O rats (*p*<0.01). The Tb.Th values were significantly higher among the SE rats than they were among S, O, OE2, and OEE rats (*p*<0.01). In addition, the OE rats showed significantly hi gher Tb.Th values than the S and O rats (*p*<0.001). The Tb.N in the SE rats was significantly higher than that of all the other rat groups (*p*<0.001). The S and OE rats showed dominantly higher Tb.N than the O rats (*p*<0.001). The Tb.Sp values of the O rats were significantly higher than those of all the other rat groups (*p*<0.001). The TBPf values of the S rats were also significantly higher than those of all the other rat groups (*p*<0.001)

**Table 4.** Trabecular parameters of the proximal tibia cancellous bone by micro-computed tomography

	S n=6		SE n=6		O n=10		OE n=11		OE2 n=6		OEE n=6	
	Mean	SE	Mean	SE	Mean	SE	Mean	SE	Mean	SE	Mean	SE
BV/TV (%)	10.26^b^	0.84	19.77^a^^c^^d^^e^^f^	3.66	4.18^b^^d^	0.29	10.92^b^^c^	1.55	7.48^b^	0.45	8.46^b^	0.39
Tb.Th (μm)	49.66^b^^d^	0.97	62.44^a^^c^^e^^f^	3.38	48.81^b^^d^	0.75	57.76^a^^c^	1.87	52.99^b^	0.91	53.27^b^	0.83
Tb.N (/mm)	2.06^b^^c^	0.15	3.06^a^^c^^d^^e^^f^	0.45	0.85^a^^b^^d^	0.05	1.84^b^^c^	0.20	1.41^b^	0.08	1.59^b^	0.06
Tb.Sp (μm)	450.74^c^	42.47	336.18^c^	105.49	1170.49^a^^b^^d^^e^^f^	89.32	535.20^c^	49.38	666.95^c^	40.82	581.38^c^	24.23
TBPf (/mm)	12.55^b^^c^^d^^e^^f^	0.07	7.70^a^	0.78	7.99^a^	0.64	6.27^a^	0.50	5.08^a^	0.70	5.11^a^	0.72

^a^p < .05 vs S

^b^p < .05 vs SE

^c^p < .05 vs O

^d^p < .05 vs OE

^e^p < .05 vs OE2

^f^p < .05 vs OEE

S, sham-operated and sedentary; SE, sham-operated and exercise trained; O, ovariectomized and sedentary; OE, ovariectomized and exercise trained; OE2, ovariectomized and given 17β-estradiol; OEE, ovariectomized, exercise trained, and given 17β-estradiol; Wt, weight

## DISCUSSION

The present study results showed a decrease in bone volume, BMD, and bone strength in O rats.

These rats also tended to have reduced Gla/Glu-osteocalcin levels, but not to a significant degree. Estrogen deficiency in postmenopausal women and ovariectomized rats increases the potential for contracting a variety of ailments, such as hot flushes, osteoporosis, and depression. Furthermore, estrogen plays a vital role in maintaining healthy bones^12^, and Katsumata *et al*. reported that ovariectomized rats have a lower bone volume, bone strength, and a faster bone metabolism turnover, a phenomenon which is also observed in women suffering from osteoporosis^13^. The decrease in bone volume, BMD, and bone strength in O rats observed in the present study is consistent with the findings of Katsumata *et al*., and it highlights the link between estrogen deficiency and decreased bone health. Estrogen deficiency is typically treated with hormone replacement therapy (HRT), which has been available for a number of decades. In this study, the OE2 rats received the equivalent of HRT, and their bone volume, BMD, and bone strength significantly improved compared with that of O rats. The positive impact of HRT observed in the present study is consistent with the findings of numerous preceding studies that report that HRT reduces the risk of bone fractures from estrogen deficiency and impedes the progression of osteoporosis^14, 15^. However, it is also reported that HRT increases the risk of contracting diseases, such as breast cancer, stroke, and cardiovascular disease. Therefore, the benefits that HRT brings to the bones may not outweigh the risks. By exercising, women can prevent bone volume reduction without resorting to drug therapies. Exercise should therefore be given the highest priority as a preventative factor.

HIST increased bone strength in the sham-operated rats and the ovariectomized rats. We did not observe any significant difference among the groups in terms of the bone metabolism index Gla/Glu-osteocalcin. Matsuda *et al*. subjected a group of female rats to jump training. One group undertook the exercise for 2 weeks, and the other for 8 weeks, and they compared the impact of such exercise on bone metabolism. The study reported that the 2-week group had low osteocalcin concentrations in their blood, while the 8-week group rats had high osteocalcin concentrations^16^. The present study subjected the rats to HIT for only 6 weeks, and so it is reasonable to assume that there was not sufficient time for the impact on osteocalcin concentration in the blood to take effect. However, with regard to tibia cancellous bone structure, we observed a significant beneficial effect among the sham-operated and ovariectomized rats, suggesting that HIST does have a beneficial effect on bones.

Turner^17^ provided 3 rules concerning bone adaptation to mechanical stress: (1) bone adaptation is driven by dynamic loading, (2) only a short duration of mechanical loading is necessary to initiate an adaptive response, and (3) bone cells accommodate to a customary mechanical loading environment. In addition, when bones respond to stress, their strength and volume changed. These changes are purely the result of the stress-response of the minimum effective strain, thus, they can be observed in the adaptive response to the stress^18^. In the present study, the rats undertook 5 HIST sessions per week, fitted with lead weighing 14–16% of their body weight. Each session consisted of 14 20-second swimming training sets with 10-second breaks in between. HIST involves high-intensity dynamic loading, and short 7-minute continuous bursts of training. As such, it cannot be considered as everyday exercise. HIST was originally designed as an exercise method for elite athletes, a method that very quickly induces an increase in the maximum oxygen intake^9^. It is likely that the beneficial effect of an increase in bone strength and improved tibia cancellous bone structure discussed in this study occurred because the HIST matched Turner’s 3 rules, and, as such, prompted intense load-induced muscular contractions as an adaptive response to loading.

We also investigated the interactive effect of HIST and HIST combined with estrogen replacement on bones. The disparity in results can probably be explained by exercise and estrogen instigating different actions in different parts of the skeleton. Yeh *et al*. examined the effects of 17β-estradiol replacement combined with treadmill exercise on the vertebral and femoral bones of ovariectomized rats. They reported that 17β-estradiol replacement had a bone-preserving effect primarily in the vertebrae, whereas treadmill exercise primarily affected the limbs^19^. In other words, the effects of estrogen therapy and those of exercise are independent and additional to each other. Furthermore, it has been reported that the impact of exercise on the proximal tibia cancellous bone is limited^20, 21^. Tromp *et al*. reported that the mechanical stress in loaded rats takes effect primarily in the cortical bone^22^. These findings suggest that, in this study, there were no significant differences between combined intervention (estrogen replacement combined with HIST) and non-combined intervention (estrogen therapy or HIST), except with regard to bone volume. However, given the similarity of the results for estrogen replacement and those for combined intervention, it is possible that the additive effect can be found more in the vertebrae than in the lower limb bones (femur and tibia), and more in the cortical bone than in the cancellous bone, suggesting that the effect varies in different parts of the skeleton. In the present study, limitation is difficult for health promotion by the wide generation because HIST is leading to fatigue. We examined an effect of high-strength intermittent swimming training (HIST) alone and in combination with an estrogen supplement for the prevention of osteoporosis. As a result, HIST was found to have a beneficial effect in terms of bone quantity and bone intensity, of the proximal tibia cancellous bone. Therefore, a strong stimulation such as HIST was suggested to also affect the bone.
